# The presentation of two rare idiopathic diseases in one patient: spontaneous idiopathic pneumoperitoneum and idiopathic gastroparesis: a case report

**DOI:** 10.1097/MS9.0000000000002170

**Published:** 2024-06-24

**Authors:** Isabela González-Acosta, Edwin L. Maldonado-García, Federico López-Jasso

**Affiliations:** a Department of Education from Beneficencia Española de la Laguna,Torreón; b Diagnostic Auxiliary Services, Beneficencia Española de la Laguna, Torreón; c Department of Surgery, Hospital General de Zona Instituto Mexicano del Seguro Social, Torreón, Coahuila, México

**Keywords:** case report, idiopathic spontaneous pneumoperitoneum, idiopatic gastroparesis, idiopathic disease

## Abstract

**Introduction:**

Idiopathic gastroparesis (GP) is a syndrome characterized by delayed gastric emptying in the absence of a mechanical obstruction and the presence of cardinal symptoms, spontaneous idiopathic pneumoperitoneum is the presence of free air in the abdomen with the exclusion of a perforated viscera on endoscopy; both diseases have a low prevalence in which no detectable primary etiology can be identified. The authors present the case of a 44-year-old female with both diseases occurring simultaneously.

**Presentation of case:**

A 44-year-old female without relevant prior medical history, presented to the emergency room with intense abdominal pain in all four quadrants, on superficial and deep palpation. Imaging studies were conducted revealing the presence of a distended stomach filled with contrast and free air in the abdomen. An endoscopy was conducted looking for an obstruction of the gastric content and a laparotomy for the presence of a perforation on the viscera; both were ruled out.

**Clinical discussion:**

Although surgical intervention is not necessary on a patient presenting either with GP or spontaneous idiopathic pneumoperitoneum, given the clinical presentation and image studies of this patient with the finding of free air in the abdomen, a distended stomach filled with contrast and severe abdominal pain, it was decided to perform a laparotomy looking for a possible source and avoid complications of a possible blockage and/or perforation in the gastrointestinal tract.

**Conclusion:**

First-time cases pose a challenge for physicians at the moment of deciding on the best treatment option for the patient, especially with low-frequency pathologies.

## Introduction

Highlights

- Spontaneous idiopathic pneumoperitoneum is a disease that should be suspected in patients who present with free air within the abdominal cavity on imaging and in who are in stable conditions.
- The most common type of gastroparesis (GP) is idiopathic GP and measurement of gastric emptying should be done in order to provide the best possible treatment for the patient.
- Expectant and symptomatic treatment should be chosen when a patient is in stable conditions, and there are no other indications for surgery.
- The publication of singular cases aids physicians in the choice of treatment for their patients if ever faced with the same case.

Gastroparesis (GP) is a syndrome composed of delayed gastric emptying in the absence of a mechanical obstruction and the presence of cardinal symptoms: early satiety, postprandial fullness, nausea, vomiting, bloating, and upper abdominal pain^1^. It most commonly affects young and middle-aged women, with 3:1 women:men ratio^2^, and according to Ye *et al*.^3^ the prevalence is 13.8 per 100 000 persons. Its physiopathology is not clearly understood, and there are discrepancies in the literature regarding its origin; nevertheless, it is believed that an impairment in gastric neuromuscular function, an intrinsic neuropathy of the stomach, and a disfunction of the immune system all have a role in the development of GP^4,5^.

Pneumoperitoneum is the presence of free air in the abdomen, most commonly due to the perforation of a hallow viscera, accounting for more than 90% of cases^6^. Idiopathic spontaneous pneumoperitoneum is diagnosed in a small sub-group of patients of the remaining 10% of cases, where all other possible causes have been ruled out, including intrathoracic, iatrogenic, gynecological, and intra-abdominal^7^.

We present the case of a young female patient who presented to the ER with abdominal pain and was diagnosed with GP and pneumoperitoneum after imaging studies. This case report has been reported in accordance with the Surgical Case Report (SCARE) 2023 criteria^8^.

## Case

A 44-year-old female patient presented to the emergency room of a tertiary care hospital with acute abdominal pain, severe intensity, located mainly to the right flank and right hypochondrium, which spread to the epigastrium, with postprandial exacerbation, fullness, associated with singultus and halitosis for the past 3 days. Her past medical history was uneventful except for 4 cesarian sections, without complications. No chronic diseases, drug, alcohol or corticosteroid consumption. She reported to be taking 3 g of Semaglutide daily for weight loss. At the physical examination in the ER, her blood pressure was 100/70 mmHg, heart rate of 73 beats per min, and body temperature of 36.2°C, pale, her white blood cell count was within the normal values. Her abdomen was normal on inspection, she had absent bowel sounds; with intense abdominal pain on superficial and deep palpation in all four quadrants, no palpation of visceromegaly.

**Figure 1:**
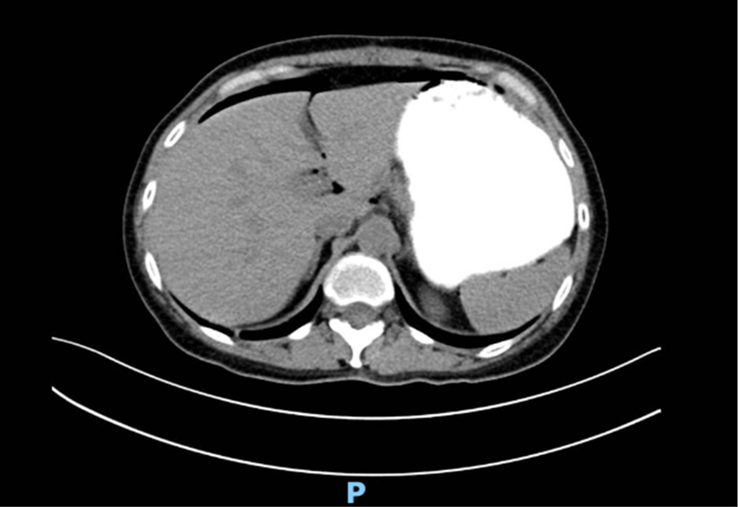
Axial computed tomography scan of the abdomen showing free peritoneal air.

**Figure 2:**
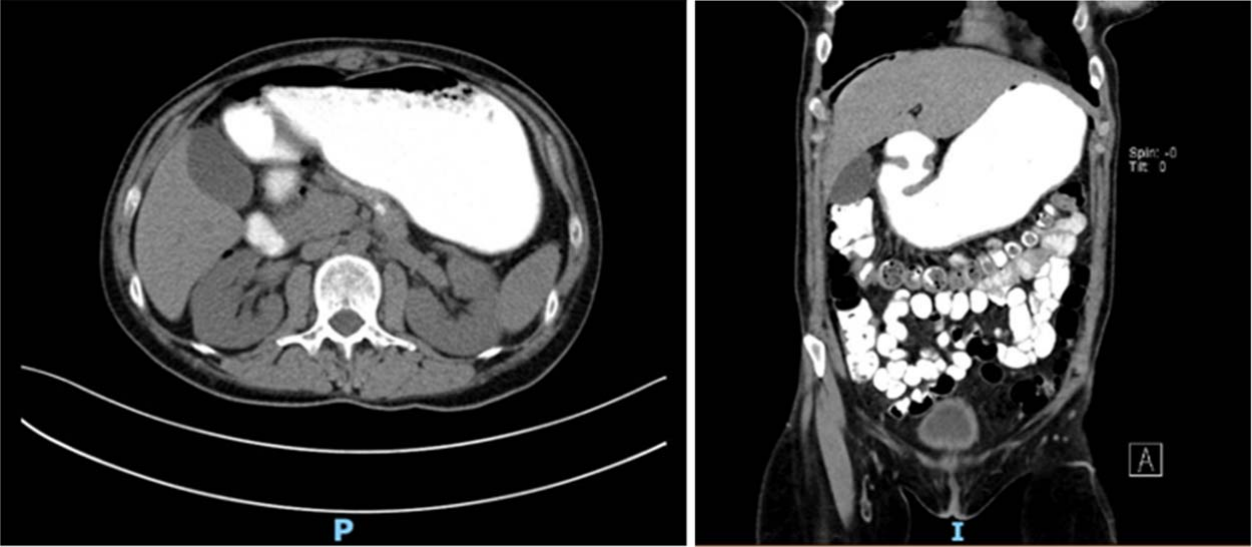
Axial (left) and coronal (right) computed tomography of the abdomen show a distended stomach filled with contrast without any evidence of leaks.

We decided to rule out an intestinal obstruction and perforation with a computed tomography (CT) scan with contrast, which revealed free peritoneal air, primarily located next to the rectus abdominis (Fig. 1), a distended stomach filled with contrast (Fig. 2), no evidence of small or large bowel obstruction or contrast leakage, and no wall thickening. There was also evidence of diverticular disease of the colon without apparent complications.

An intravenous access was obtained to administer analgesia and third-generation cephalosporins. However, the patient continued with symtpoms of acute abdomen, intense pain, and tachycardia, regardless of treatment.

An upper endoscopy and exploratory laparotomy (due to the unavailability of laparoscopy at the moment) were performed looking for a source of the patient’s findings, to rule out a mechanical blockage explaining the delayed gastric emptying and a gastrointestinal perforation as the etiology of the peritoneal free air. No definitive causes were identified. Additional tests were ordered—including antibodies for Systemic Scleroderma—to rule out other sources of GP, all with negative results. A nasogastric tube was placed to empty the stomach content and symptomatic treatment with third-generation cephalosporins and analgesics was given until the improvement of her symptoms and her discharge on postoperative day 6 without further complications.

## Discussion

GP is a disease of unknown origin, characterized by delayed gastric emptying in the absence of mechanical obstruction and the presence of cardinal symptoms, including early satiety, postprandial fullness, nausea, vomiting, bloating, and upper abdominal pain^1^, most of which were reported by our patient. After the exclusion of a mechanical obstruction by an upper gastrointestinal (GI) endoscopy, diverse tests can be made to assess the motility and function of the stomach in patients who present with GP^4^. Scintigraphy GE (SGE) is the gold standard for the measurement of gastric emptying^1^, needed to confirm and identify the severity of the disease, and choose the appropriate treatment.

Once the diagnosis has been established, the etiology of GP should be identified to treat any underlying cause. Several sources have been documented, the most important ones being: diabetes mellitus (type 1 and 2), iatrogenic, and idiopathic. This case concurs with McCallum and colleagues who reported that the most common etiology of GP is idiopathic—in which no detectable primary underlying abnormality can be found—with 36% of cases studied described as such^9^. Recently, the use of Semaglutide has been described as a possible source of GP in patients who take the antidiabetic; however, further research is needed to prove this hypothesis, which is why we could not determine its use as the source of GP^10^.

Management of GP is dependent on the degree of gastric emptying delay as assessed by SGE and will be adjusted depending on severity. The main regime focuses on correcting fluid, electrolyte, and nutritional deficiencies, identifying and treating the underlying cause, relief of symptoms and the improvement of gastric emptying^11^, which are adjusted to each specific case.

Pneumoperitoneum is the presence of free air in the abdomen due to the perforation of a viscera; its finding is always abnormal. Perforation represents the majority of cases of pneumoperitoneum, of which approximately 90% are attributable to gastric or duodenal perforations; however, other sources can be identified^12^. Spontaneous pneumoperitoneum (Table 1)^12,13^ is determined by the absence of a perforation in the gastrointestinal tract, it accounts for the remaining 10% of cases. Idiopathic spontaneous pneumoperitoneum, where no source of the free intra-abdominal air is identified, is an exclusion diagnosis, and accounts for the smallest portion of cases of spontaneous pneumoperitoneum^6^.

**Table 1:** Causes of spontaneous pneumoperitoneum adapted from Mann *et al*.^12^ and Camilo-Cardona *et al*.^13^.

Rupture hollow viscus
Infection of the peritoneal cavity with gas forming bacteria
Rupture of a thoracic abscess
Iatrogenic causes
Bowel obstruction
Pneumatosis intestinalis
Vaginal warm showers, postpartum, postcoital state
Chemoembolization of liver tumors
Pneumomediastinum, pneumothorax and bronchopleural fistula
Cardiopulmonary resuscitation
Mechanical ventilation
Idiopathic bowel perforation
Idiopathic

The most common presenting symptom is abdominal pain with unspecific characteristics, a very common presenting symptom in the emergency department. Most of these patients will undergo a chest X-ray looking for a source of pain, which reveals a radiolucent image inferior to the diaphragm, representing the free air within the abdominal cavity^13^. This finding is characteristic of pneumoperitoneum, and is highly suggestive of a perforated abdominal viscus, which is a surgical emergency. However, when the patient is in stable condition, careful evaluation is indicated, along with a thorough medical history, blood tests, and the current state of the patient to opt for surgical intervention. In the absence of peritonitis and sepsis, surgery should be withheld to avoid an unnecessary procedure. As recorded by Mularski *et al.* ^6^ from a systematic review of the literature in which, out of 196 case reports of non-surgical pneumoperitoneum, 45 involved surgical exploration without any evidence of a perforated viscus, and in which, as seen retrospectively, surgery could have been avoided.

Unlike pneumoperitoneum due to the perforation of a viscera, most cases of spontaneous pneumoperitoneum can be managed with conventional treatment, consisting of IV fluids, antibiotics, and observation for worsening symptoms and/or blood tests^14^. No guidelines have been published regarding the management of idiopathic spontaneous pneumoperitoneum; however, some authors have reported their management strategies for these cases. In a study by Udelsam *et al*.^15^ it was found that nonoperative management of pneumoperitoneum in the absence of peritonitis was associated with similar mortality, but with reduced morbidity compared with the operative treatment group. Camilo-Cardona *et al.* ^13^ concluded that expectant management and laboratory/imaging studies are prudent in cases in which there are no signs of peritoneal irritation in patients who are in good general state. Finally, according to Mann *et al.* ^12^ the decisions based on an accurate and thorough medical history and physical examination provide the most appropriate management options, making the patient’s clinical condition the most important factor to guide treatment. Given that our patient presented to the emergency room with severe abdominal pain, the posterior finding of pneumoperitoneum, and a distended stomach filled with contrast on imaging studies, a more conservative treatment could not be carried out due to the potential complications of a blockage to the stomach and perforation of the viscera, considered due to her presentation and imaging results. The simultaneous occurrence of these two pathologies justified the otherwise non-surgical treatment of this patient. In retrospect, the patient could have been managed with conservative treatment alone; however, her unique clinical presentation prompted for a more aggressive strategy at the time.

To our knowledge, this could be the first case report to document the presentation of spontaneous idiopathic pneumoperitoneum and idiopathic GP simultaneously in a patient.

## Conclusion

The presentation of uncommon pathologies poses a challenge for physicians when deciding on a treatment course, especially when several pathologies are simultaneously present. A careful evaluation of the patient and review of the current literature should be done to guarantee a necessary surgical procedure; however, cases like this one force physicians to forgo from current recommendations and choose an alternative treatment. Only when seen retrospectively can physicians ascertain whether the right decision was made, making the diffusion of “special” cases indispensable to aid physicians who are presented with the same problem in the future.

## Ethical approval

Ethical approval is not required.

## Consent

Written informed consent was obtained from the patient for publication of this case report and accompanying images. A copy of the written consent is available for review by the Editor-in-Chief of this journal on request.

## Source of funding

The authors declared that this study received no financial support.

## Author contribution

All authors read and approved the final manuscript. I.G.-A.: conceptualization, investigation, visualization, writing—original draft. E.M.-G.: conceptualization and writing—review and editing. F.L.-J.: resources, supervision, writing—review and editing.

## Conflicts of interest disclosure

This manuscript has not been submitted to, nor is it under review at, another journal or other publishing venue. The authors have no affiliation with any organization with a direct or indirect financial interest in the subject matter discussed in the manuscript.

## Research registration unique identifying number (UIN)

Not applicable.

## Guarantor

Isabela Gonzalez-Acosta and Dr Edwin L. Maldonado-Garcia.

## Data availability statement

Data sharing is not applicable to this article.

## Provenance and peer review

No commissioned, externally peer-reviewed.
